# Impact of Chronic Spontaneous Urticaria on Major Life-Changing Decisions: An Exploratory Cross-Sectional Study

**DOI:** 10.3390/jcm15187222

**Published:** 2026-09-17

**Authors:** Lucía Fuentes-Barragán, Francisco Javier León-Pérez, Raquel Sanabria-de la Torre, Carmen García-Moronta, Alejandro Molina-Leyva, Salvador Arias-Santiago, Trinidad Montero-Vílchez

**Affiliations:** 1 Department of Biochemistry and Molecular Biology III and Immunology, University of Granada, 18016 Granada, Spain; luciafuentes@correo.ugr.es (L.F.-B.); raquelsanabriadlt@gmail.com (R.S.-d.l.T.); 2 Department of Dermatology, University Hospital Virgen de las Nieves, 18014 Granada, Spain; marcarpera@gmail.com (F.J.L.-P.); carmengmoronta@gmail.com (C.G.-M.); alejandromolinaleyva@gmail.com (A.M.-L.); tmonterov@gmail.com (T.M.-V.); 3 Biosanitary Research Institute of Granada, 18014 Granada, Spain

**Keywords:** Chronic Spontaneous Urticaria, major life-changing decisions, quality of life

## Abstract

**Background:** Chronic Spontaneous Urticaria (CSU) profoundly impairs health-related quality of life due to intense pruritus and unpredictable flares. However, its specific influence on patients’ major life-changing decisions (MLCD)—such as career choices, family planning, and personal relationships—remains under-researched. This study aimed to evaluate the overall impact of CSU on MLCD and analyze how disease severity modifies these fundamental life trajectories. **Methods:** This exploratory, cross-sectional study included adult patients with active CSU (≥6 months’ duration) consecutively recruited from a tertiary hospital. Clinical data were collected, and disease activity was captured via the validated patient-reported Urticaria Activity Score over 7 days (UAS7), categorizing patients as having severe (UAS7 ≥ 28) or mild-to-moderate (UAS7 < 28) disease activity. A 4-point Likert scale adapted from the conceptual framework by Bhatti et al. was used to assess the perceived impact across multiple life-changing decision domains. **Results:** Thirty patients were evaluated (mean duration 5.6 years; mean UAS7 30.47). CSU substantially impacted MLCD, particularly lifestyle habits (70%), work performance (63.3%), domestic travel/leisure (60%), and clothing choices (60%). Patients with severe CSU reported a markedly higher impairment in professional career choices (52.6% vs. 25.0%, *p* = 0.030), work performance (84.2% vs. 37.5%, *p* = 0.036), educational attainment (25.0% vs. 0.0%, *p* = 0.038), family relationships (71.5% vs. 11.1%, *p* = 0.022), and lifestyle habits (90.5% vs. 22.2%, *p* = 0.002) compared to those with mild-to-moderate disease. **Conclusions:** In this exploratory cohort, CSU was associated with substantial impairments in patients’ MLCD, with a higher frequency of disruption observed among those with severe disease activity. These findings highlight the value of adopting a biopsychosocial perspective in clinical practice to support life trajectories alongside cutaneous symptom control.

## 1. Introduction

Chronic Spontaneous Urticaria (CSU) is a debilitating cutaneous condition driven by dysregulated mast cell activation, clinically characterized by the recurrent appearance of transient wheals, angioedema, or both, for a duration of at least six weeks [1]. Typical wheals present as a central swelling surrounded by reflex erythema, associated with intense pruritus or a burning sensation, and usually resolve within 24 h [1]. Although its complex pathogenesis may involve various infectious, pseudo-allergic, or autoimmune factors, the underlying cause remains idiopathic in a high percentage of cases, ranging between 80% and 90% in clinical practice [1,2,3].

In Spain, CSU represents a substantial epidemiological burden with an estimated prevalence of 0.6%, showing a growing trend in recent years within European healthcare registries [4]. Despite its low mortality, CSU exerts a profoundly negative impact on patients’ health-related quality of life (HRQoL) [1]. The clinical features of the disease—particularly the severe pruritus and the high unpredictability of acute flares—disrupt numerous daily activities by interfering with sleep architecture, work or academic performance, and social interactions [4]. Indeed, the overall impairment in HRQoL reported by this population has been described as comparable, or even superior, to that caused by other severe chronic systemic conditions, such as advanced ischemic heart disease [4,5,6].

Importantly, the consequences of CSU may extend beyond its immediate effects on daily functioning and influence major decisions with long-term personal and professional implications. Throughout an individual’s life trajectory, pivotal moments requiring decisions that shape their future professional and personal development; these are conceptualized as major life-changing decisions (MLCD) [7,8]. The diagnosis and daily experience of living with a chronic, unpredictable condition like CSU can profoundly influence these choices, compelling patients to continuously reassess their priorities, expectations, and long-term goals. These fundamental MLCD span multiple biographic domains, including the choice of a professional or educational career, family and partner relationships, reproductive choices, and sexuality, as well as housing logistics, leisure activities, sports, and daily lifestyle habits [7,8].

The influence of chronic illness on MLCD was initially conceptualized and evaluated through dedicated instruments by Bhatti et al. [7,8]. To date, the negative impact of high-burden dermatological diseases such as cancer and psoriasis on patients’ MLCD has been objectively documented in the literature [7,9]. Despite the high prevalence of CSU and its well-established disruption of standard quality-of-life indices [4], there is a notable paucity of data regarding how this specific mast-cell-driven disease affects the making of MLCD.

Understanding this influence is fundamental to transitioning from a purely symptomatic therapeutic model to a comprehensive, tailored biopsychosocial approach that meets the real long-term needs of these patients. Therefore, the primary objective of this study was to evaluate the impact of CSU on the making of major life decisions. The secondary objective was to investigate how this biographical impact differed according to patient-reported disease activity assessed via the UAS7.

## 2. Materials and Methods

### 2.1. Study Design and Setting

This exploratory, cross-sectional, descriptive, and observational study was conducted at the outpatient clinics of the Department of Dermatology at the Hospital Universitario Virgen de las Nieves (Granada, Spain), a tertiary care referral center. Patient recruitment was performed consecutively during scheduled follow-up appointments.

### 2.2. Patient Selection and Eligibility Criteria

The study population consisted of adult patients diagnosed with CSU. To ensure focused evaluation and minimize confounding factors linked to other chronic morbidities, eligibility criteria were applied:

- Inclusion Criteria: (1) Clinical diagnosis of CSU with a documented disease duration of at least 6 months; (2) age over 18 years; and (3) provision of written informed consent prior to enrollment.
- Exclusion Criteria: (1) Isolated inducible chronic urticaria without spontaneous wheals (e.g., symptomatic dermographism, cholinergic or cold urticaria); (2) concomitant chronic inflammatory skin diseases with a known high burden on quality of life (e.g., psoriasis, hidradenitis suppurativa, atopic dermatitis); (3) any severe systemic chronic disease capable of independently conditioning life choices (e.g., severe cardiovascular disease, poorly controlled diabetes mellitus, or history of solid organ transplantation); (4) active malignancy or a history of cancer within the past 5 years; and (5) explicit refusal to participate in the study.

### 2.3. Variables and Clinical Measurement Instruments

Data collection was structured into three main axes through clinical interviews, physical examinations, and validated self-administered questionnaires:

#### 2.3.1. Sociodemographic and Baseline Clinical Characteristics

Sociodemographic and biometric variables included age, sex, current employment status, and highest educational level attained. Clinical parameters evaluated comprised age at CSU onset, overall disease duration, smoking habits, number of affected body areas, and relevant comorbidities (including sleep disturbances, anxiety, and depression). Topographical distribution was recorded based on the clinical involvement of anatomical regions such as the face, trunk and limbs, inframammary fold, hands, buttocks and genitalia.

#### 2.3.2. Evaluation of Major Life-Changing Decisions (MLCD)

The impact of CSU on pivotal biographic decisions was quantified using a specialized structured questionnaire adapted from the conceptual framework developed by Bhatti et al. Individual life decisions were rated by patients using a 4-point Likert scale ranging from 0 (“No impact”) to 3 (“Significant/Much impact”) (see Appendix A). The instrument explored 26 specific life-changing decisions grouped into seven distinct clinical-biographical domains:

1. Work-related decisions: Career choice, job performance, promotion opportunities, work absenteeism, income/salary, disease-driven job loss, and early retirement.
2. Education-related decisions: Academic performance and achievement of the desired educational level.
3. Personal relationships: Family dynamics, socialization/friendships, romantic life, and difficulty finding a partner.
4. Reproductive desires and sexuality: Desire to have children, decision to remain childless, restriction on the desired number of children, and presence of sexual dysfunction.
5. Housing and relocation: Choice of habitual residence, geographic location of the home, and the decision to live abroad.
6. Vacations and leisure: Frequency and destination choices for national and international leisure trips.
7. Lifestyle and substance use: Daily lifestyle habits, clothing selection, sports participation, smoking, alcohol consumption, and the requirement for psychopharmacological therapy (anxiolytics, antidepressants, or hypnotics).

Additionally, the questionnaire evaluated the specific negative burden of key physical and emotional symptoms (pain, pruritus, sleep deprivation, physical limitation, embarrassment, lack of confidence, stress/anxiety, and sadness/depression) using the same 4-point scale.

#### 2.3.3. Disease Severity and Quality of Life Indices

- Patient-Reported Outcomes (PROMs): The Urticaria Activity Score over 7 days (UAS7) was used to measure disease activity. The UAS7 is a well-established, validated patient-reported outcome measure (PROM) based on the prospective daily recording of wheal numbers (0 to 3) and pruritus intensity (0 to 3) over 7 consecutive days, yielding a cumulative score from 0 to 42. To analyze the clinical impact of severity on MLCD, patients were stratified into two cohorts: mild-to-moderate disease activity (UAS7 < 28) and severe disease activity (UAS7 ≥ 28).
- Health-related quality of life was measured using the Dermatology Life Quality Index (DLQI), a 10-item validated instrument with a cumulative score from 0 to 30. Subjective symptom intensity over the preceding week was captured via an 11-point Numerical Rating Scale (NRS) for pruritus (0 = no itch; 10 = worst imaginable itch) and an NRS for sleep disturbance (0 = no impact; 10 = critical sleep disruption). Finally, the global perceived long-term biographical impact of CSU was self-reported by patients on a 5-point categorical scale (Absent, Minimal, Moderate, High, or Very High).

### 2.4. Statistical Analysis

Descriptive statistics were applied to summarize sample characteristics. Continuous variables were tested for normality using the Shapiro–Wilk test and expressed as mean ± standard deviation (SD) for normally distributed data, or as median and interquartile range (IQR) for non-parametric variables. Categorical variables were expressed as absolute frequencies (*n*) and relative percentages (%).

Comparative analyses between clinical cohorts were performed based on data distribution:

- To compare nominal variables, the chi-squared (χ^2^) test or Fisher’s exact test was used, as appropriate.
- To compare nominal with continuous variables, Student’s *t*-test or the Wilcoxon–Mann–Whitney test was used, depending on the normality of the data and the nature of the variables.

Exploration of relationships and associated factors:

- Pearson’s correlation coefficient was used to evaluate the relationship between the different continuous variables.
- The exploration of relationships between variables and the quantification of the magnitudes of association were carried out using linear regression analysis. The independent variables (disease severity, sex, age) were defined as those considered to be predictors of, or influences on, the dependent variable (the making of MLCD).

A two-tailed *p*-value < 0.05 was considered statistically significant. All statistical analyses were performed using SPSS version 24.0 (SPSS Inc., Chicago, IL, USA).

### 2.5. Ethical Considerations

The study protocol was reviewed and formally approved by the Biomedical Research Ethics Committee of Granada (Andalusian Biomedical Research Ethics Portal, reference: 1422-N-23). The study was conducted in strict compliance with the ethical principles of the Declaration of Helsinki. All participants (or their legal representatives) received an information sheet and provided written informed consent prior to any study-related procedures. Patient confidentiality was strictly protected through data pseudo-anonymization in accordance with the Spanish Organic Law 3/2018 on Personal Data Protection.

## 3. Results

### 3.1. Sociodemographic and Baseline Clinical Characteristics of the Cohort

A total of 30 patients with CSU were included in the study. The mean age was 46.3 years (standard deviation [SD] 14.65), and the female-to-male ratio was 3.28:1 (23 women and 7 men).

The majority of patients experienced disease onset in adulthood (mean age of onset: 40.7 years, SD 16.35) and had a mean CSU duration of 5.6 years (SD 9.96). Most patients were professionally active (76.7%, 23/30), non-smokers (63.3%, 19/30), and had attained higher education (66.7%, 20/30). Regarding reported comorbidities, sleep disturbances (40%, 12/30), anxiety (26.7%, 8/30), and concomitant atopic dermatitis (23.3%, 7/30) were prominent. The mean number of affected body areas was 4.5 (SD 1.59), with the most frequently involved sites being the trunk and limbs (96.7%, 29/30), the face (66.7%, 20/30) and the buttocks (60%, 18/30).

In terms of severity, the mean UAS7 score was 30.47 (SD 12.88), indicating a high overall disease activity in the sample. The mean impact on quality of life, as measured by the DLQI, was 14.3 (SD 7.47). The remaining demographic characteristics are summarised in Table 1.

### 3.2. Bivariate Correlations Between Objective Severity and Patient-Reported Outcomes

**Figure 1:**
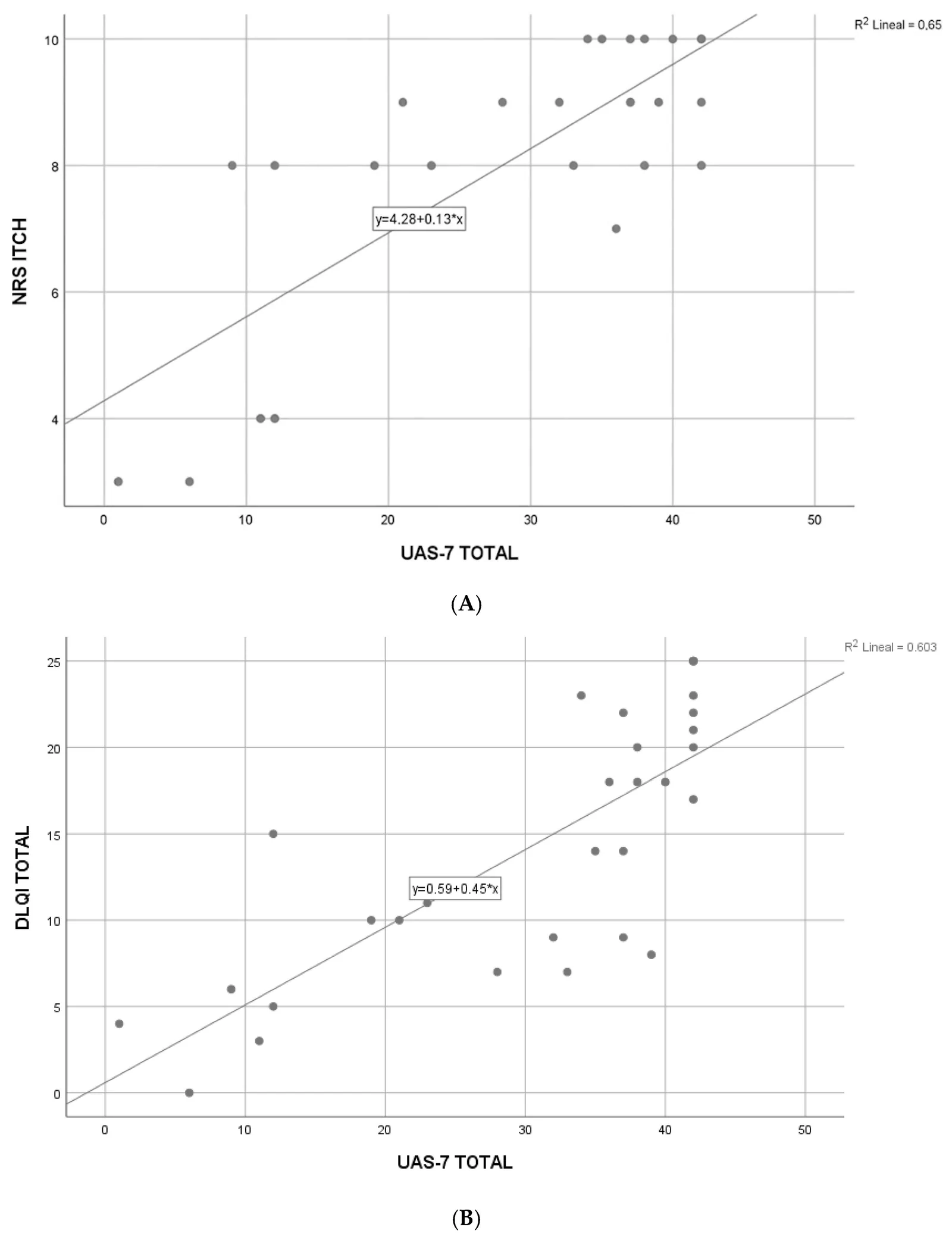

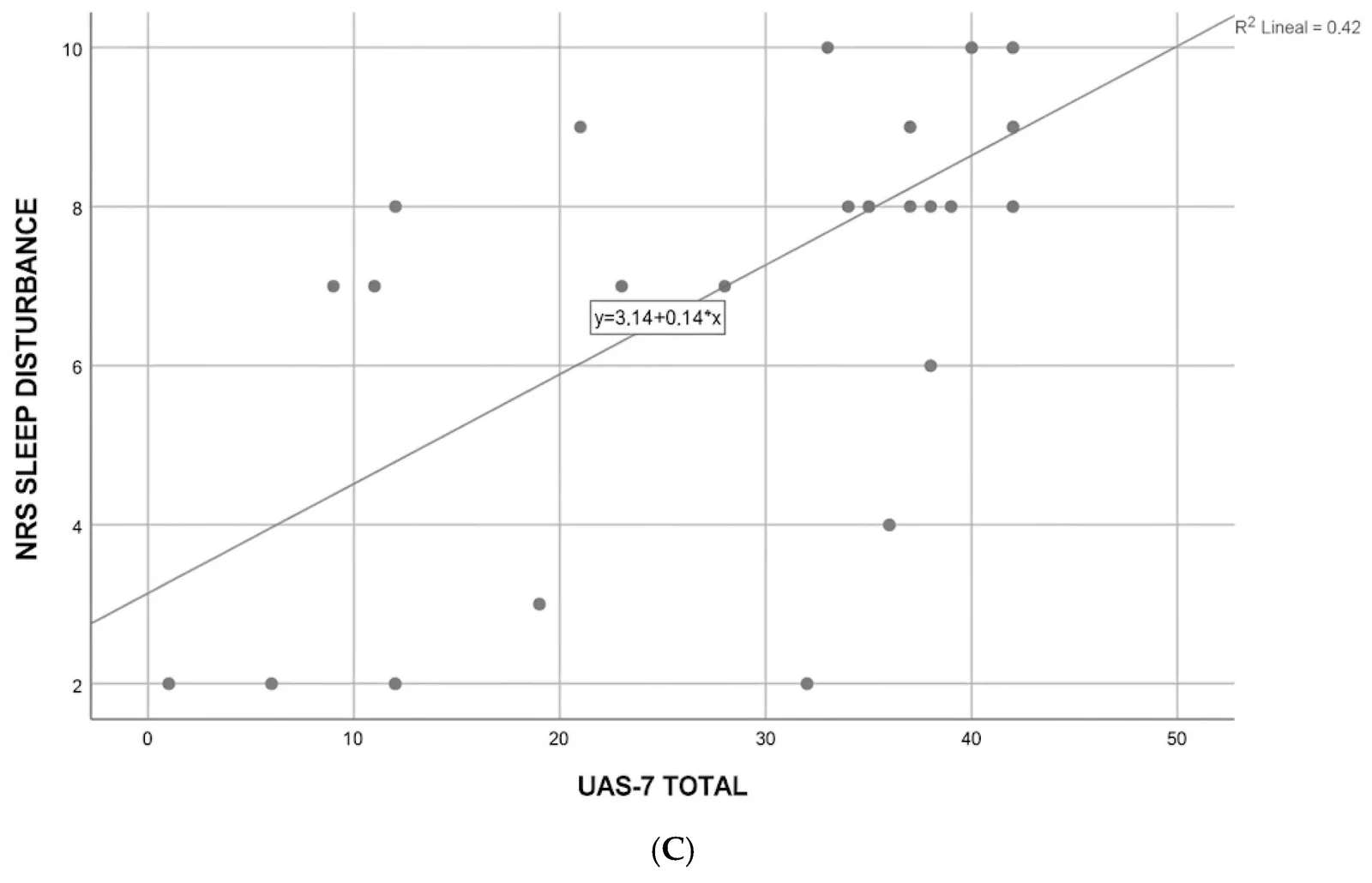
Bivariate correlation analysis between disease activity (total UAS7 score) and patient-reported outcome measures in patients with CSU: (**A**) Numerical Rating Scale (NRS) for itch (r = 0.807, *p* < 0.001); (**B**) total Dermatology Life Quality Index (DLQI) score (r = 0.776, *p* < 0.001); and (**C**) NRS for sleep disturbance (r = 0.655, *p* < 0.001).

Bivariate correlation analysis demonstrated a positive and statistically significant association between disease activity (total UAS7 score) and patient-reported outcome measures (PROMs). Specifically, higher UAS7 scores correlated with increased NRS pruritus intensity (r = 0.807, *p* < 0.001) (Figure 1A) and profound impairment of health-related quality of life measured via total DLQI scores (r = 0.776, *p* < 0.001) (Figure 1B). A significant positive correlation was also observed between UAS7 and the NRS for sleep disturbance (r = 0.655, *p* < 0.001) (Figure 1C). While a strong correlation between the UAS7 and NRS pruritus was inherently expected—given that daily itch severity represents an intrinsic component of the UAS7 score—this finding provides valuable internal validation confirming the reliability and consistency of symptom reporting within our cohort.

### 3.3. Quantifying the Global Impact of CSU on Major Life-Changing Decisions

**Figure 2:**
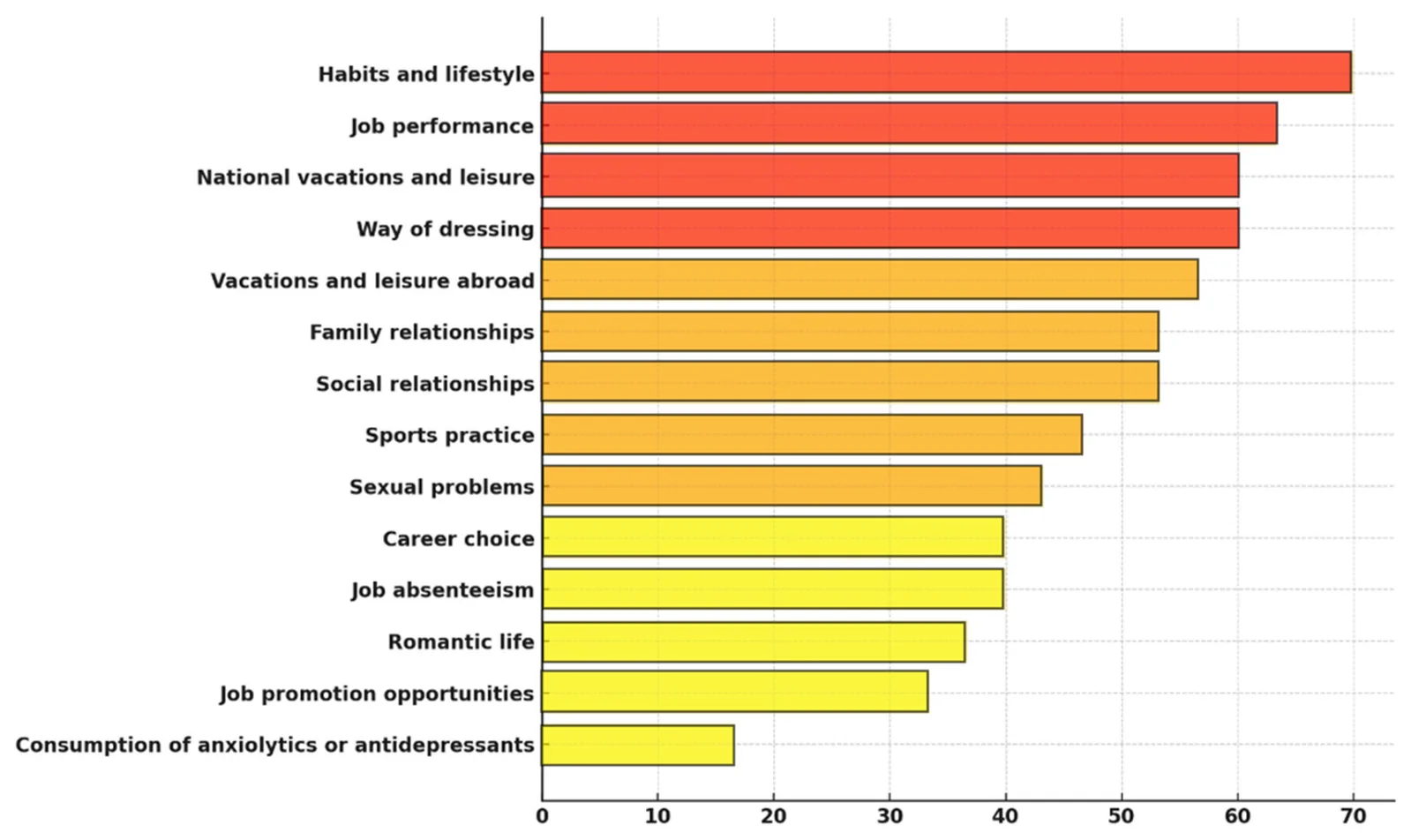
Percentage of patients with CSU reporting a moderate-to-severe impact on the most affected major life-changing decisions (MLCD). Bar colors illustrate the gradient of perceived impact frequency (red: highest impact ≥ 60%; orange: intermediate impact 40–59%; yellow: lower impact < 40%).

An impact of CSU on the making of major life-changing decisions was observed. A moderate-to-severe impact on overall lifestyle was reported by 70.0% of patients. Other frequently impaired domains included job performance (63.3%), domestic travel and leisure (60.0%), clothing choices (60.0%), international travel (56.7%), family relationships (53.3%), social relationships (53.3%), sports participation (46.7%), sexual relationships (43.3%), choice of professional career (40%), work absenteeism (40%), romantic life (36.7%), career promotion opportunities (33.4%), and the use of anxiolytics and antidepressants (16.6%). Table 2, Figure 2.

MLCD were also substantially conditioned by physical and emotional symptoms related to CSU, including pain, pruritus, sleep deprivation, physical limitation, embarrassment, stress/anxiety, and sadness/depression.

### 3.4. Stratified Analysis of MLCD Impairment by CSU Disease Activity

To determine the specific influence of disease activity on biographical trajectories, the sample was stratified into a mild-to-moderate cohort (UAS7 < 28, *n* = 9) and a severe cohort (UAS7 >= 28, *n* = 21). Comparative analysis revealed that patients with severe disease activity experienced a significantly greater impairment across multiple pivotal life choices.

**Figure 3:**
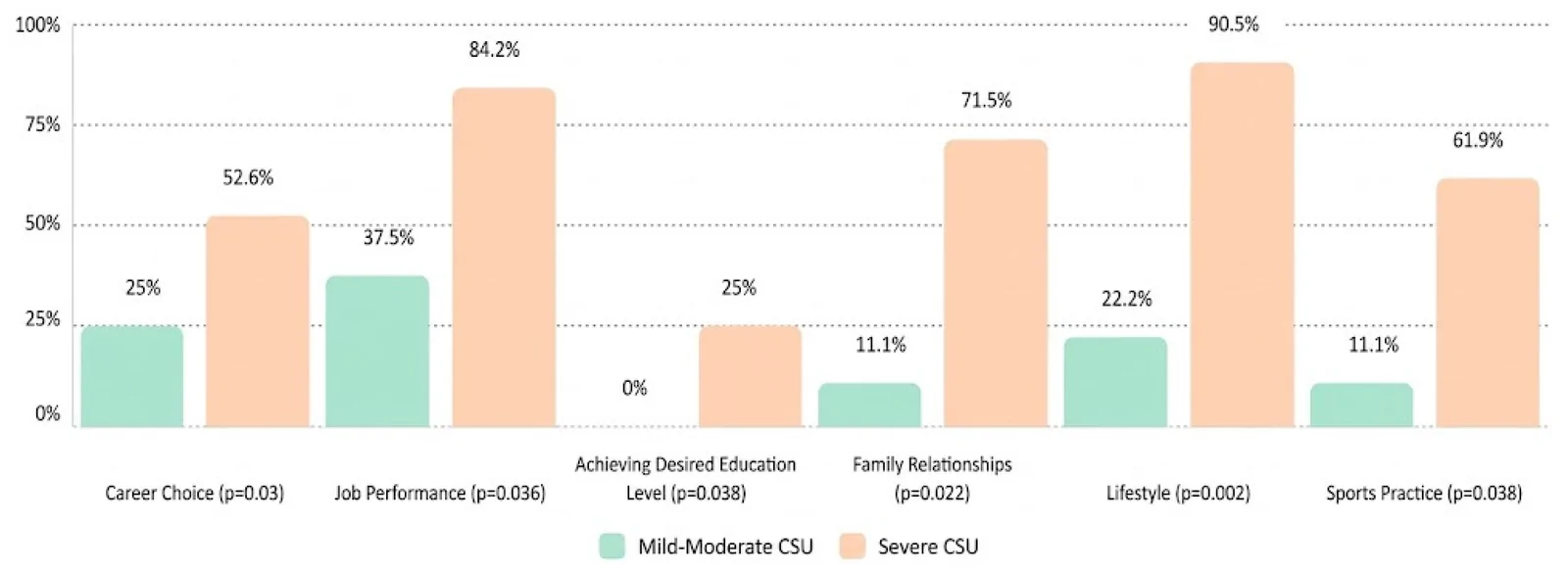
Percentage of patients with moderate–severe impact on selected life-changing decisions, stratified by CSU disease activity. Only decisions with statistically significant differences (*p* < 0.05) are shown.

Statistically significant differences were established in professional career choice (52.6% severe vs. 25.0% mild-to-moderate, *p* = 0.030), job performance (84.2% vs. 37.5%, *p* = 0.036), and the restricted capability to achieve the desired educational attainment level (25.0% vs. 0.0%, *p* = 0.038). Furthermore, severe disease status was strongly associated with deteriorated family relationships (71.5% vs. 11.1%, *p* = 0.022), forced modification of daily lifestyle habits (90.5% vs. 22.2%, *p* = 0.002), and significant limitation in regular sports participation (61.9% vs. 11.1%, *p* = 0.038). Decisions with statistically significant differences are shown in Figure 3.

Similarly, severe clinical activity was associated with a significantly higher burden across several physical and emotional symptoms, demonstrating positive associations with reported pain (*p* = 0.003), pruritus (*p* = 0.001), severe sleep deprivation (*p* = 0.002), physical fatigue (*p* = 0.002), social embarrassment (*p* = 0.010), stress/mood swings (*p* = 0.010), and reactive sadness/depression (*p* = 0.008). The comprehensive statistical stratification of sociodemographic data, clinical variables, and specific MLCD by UAS7 classification is detailed systematically in Table 3.

## 4. Discussion

The present study indicates that CSU exerts a considerable, moderate-to-severe impact on MLCD. Specifically, a significant influence was observed in domains such as lifestyle, work performance, holiday and leisure planning, choice of clothing, and social, family, and sexual relationships, among others. Furthermore, patients with greater disease severity, assessed by the UAS7, experience an even more pronounced impact on these life-changing decisions.

These results are consistent with and expand upon existing evidence regarding impaired quality of life in patients with CSU [4,10]. The literature consistently highlights that factors such as intense pruritus, associated sleep disturbance [6,11], and unpredictable disease flares constitute major drivers of the overall disease burden [10]. These physical manifestations induce substantial psychological distress, including social embarrassment, fatigue, anxiety, and depressive symptoms [12,13], which ultimately hamper social, occupational, and academic functioning [4,10]. Our study confirms this multidimensional impairment by objectifying the impact on specific MLCD related to these domains. Additionally, the strong correlation observed between UAS7 and pruritus NRS (r = 0.807), while inherently expected due to construct collinearity, serves as an internal check supporting the consistency of patients’ symptom self-assessment alongside sleep disruption and DLQI scores.

Likewise, the observation that the impact on MLCD is greater in patients with more severe CSU (UAS7 ≥ 28) is in line with previous studies that correlate higher UAS7 scores with poorer quality of life outcomes [4]. Indeed, CSU severity has been shown to be an independent predictor of quality of life [12]. Our findings reinforce this association, showing how specific decisions in the professional, educational, and social domains are more affected in patients with severe disease.

Our findings align with previous evidence evaluating the impact on MLCD in other chronic inflammatory skin diseases, such as psoriasis [9] and atopic dermatitis [14,15,16]. These studies highlighted major disruptions in career choice, work performance, absenteeism, and clothing preference [9,16]. This convergence across CSU, psoriasis, and atopic dermatitis is expected, as they share key clinical features—including chronic pruritus, visible lesions, and associated stigmatization—that collectively constrain long-term life decisions in a similar manner.

To our knowledge, this is the first study to specifically evaluate the impact of CSU on patients’ major life-changing decisions. Future studies on other chronic dermatological diseases could benefit from using specifically validated questionnaires to measure the impact on MLCD, such as the Major Life Changing Decision Profile (MLCDP) developed by Bhatti et al. [7,8], to standardise the assessment and allow for more robust comparisons. The evaluation of the impact on MLCD could even be considered an additional measure of the overall burden of disease in clinical practice.

An interesting finding in our demographic comparison was the significant difference in disease duration between cohorts, with patients in the mild-to-moderate group presenting a longer mean history of CSU than those in the severe group (11.22 vs. 3.19 years, *p* = 0.041). This disparity likely reflects patient selection dynamics inherent to tertiary referral centers. Patients with shorter disease duration presenting with acute, highly active, or poorly controlled symptoms are more prone to seek specialized hospital care during severe flares. In contrast, individuals living with CSU for more than a decade may have developed psychological habituation, disease acceptance, or practical coping strategies over time, which could attenuate perceived daily disease activity scores. Nevertheless, we cannot exclude the possibility that disease chronicity itself, independent of immediate flare activity, may exert a cumulative influence on biographical trajectories. Further longitudinal studies are needed to disentangle the distinct contributions of acute symptom severity versus long-term disease exposure to life-changing choices.

This study has certain limitations that should be considered. Firstly, its cross-sectional design precludes establishing causal relationships between CSU activity and major life decisions, meaning findings must be interpreted strictly as observational associations. Secondly, the sample size of 30 patients, particularly the numerical imbalance between the mild-to-moderate and severe CSU cohorts (*n* = 9 vs. *n* = 21), represents a major limitation that restricts statistical power and increases the risk of type II errors. Consequently, all between-group analyses must be interpreted as exploratory and hypothesis-generating rather than robust or generalizable differences. Thirdly, conducting the study in a tertiary hospital outpatient setting may have resulted in an overrepresentation of severe or refractory cases. Additionally, regarding pharmacological therapies, 14 of the 30 patients reported treatment with omalizumab, while the remaining patients were receiving H1-antihistamines. However, treatment exposure was not prospectively collected as a structured study variable, and detailed information regarding treatment duration, dosage, therapeutic response, or active status at the time of questionnaire completion was not systematically recorded. Therefore, reliable stratified comparisons according to biologic treatment could not be performed. Biologic treatment may act as a potential confounder, as achieving disease control could attenuate patients’ current perception of disease burden, while patients prescribed biologics may simultaneously represent a subpopulation with historically more severe or persistent disease. Future prospective multicenter studies should systematically capture detailed treatment histories and evaluate their modulating role on major life-changing decisions.

In conclusion, this exploratory study suggests that major life-changing decisions represent an important, often overlooked dimension of the global burden in patients with CSU. Higher disease activity was associated with more pronounced disruptions across occupational, educational, family, and lifestyle domains. These observations underscore the clinical need to integrate a comprehensive biopsychosocial approach into routine practice, supporting patients’ long-term biographic trajectories beyond cutaneous symptom control, as the true burden of the disease extends well into their psychological, emotional, and social spheres.

## Figures and Tables

**Table 1:** Socio-demographic and clinical characteristics of the CSU population.

Socio-Demographic and Clinical Characteristics (*n* = 30)				
Demographic characteristics			Disease characteristics	
Age, mean (SD)		46.3 (14.65)	Age at disease onset, mean (SD)	40.70 (16.35)
Sex, % (*n*)			Disease duration, mean (SD)	5.6 (9.96)
	Female	76.7 (23)	Number of affected areas, mean (SD)	4.5 (1.59)
	Male	23.3 (7)	Affected areas, % (*n*)	
Education level, % (*n*)			Face	66.6 (20)
	Higher education	66.7 (20)	Trunk and limbs	96.7 (29)
	Basic education	33.3 (10)	Inframammary	36.7 (11)
	None	0 (0)	Hands	56.7 (17)
Employment status, % (*n*)			Buttocks	60 (18)
	Active	76.7 (23)	Genitalia	36.7 (11)
	Student	6.7 (2)	Disease severity	
	No	63.3 (19)	NRS itch, mean (SD)	8.33 (2.12)
	Yes	36.7 (11)	NRS sleep, mean (SD)	7.33 (2.71)
Comorbidities, % (*n*)			DLQI, mean (SD)	14.3 (7.47)
	Sleep disorders	40 (12)	UAS 7, mean (SD)	30.47 (12.88)
	Asthma	20 (6)		
	Fatigue/Low performance	20 (6)		
	Depression	16.7 (5)		
	Rhinoconjunctivitis	16.7 (5)		

Values are expressed as mean ± standard deviation (SD) for continuous variables and relative (%) and absolute frequencies (*n*) for categorical variables.

**Table 2:** Mean score of each MLCD studied in patients with CSU.

Life Decisions Evaluated, Median (IQR)		
Life decisions related to work		
	Impact of CSU on career choice	1 (0–3)
	Impact of CSU on job performance	2 (1–3)
	Impact of CSU on promotion opportunities	1 (0–3)
	Impact of CSU on job absenteeism	1 (1–3)
	Impact of CSU on income and salary	0 (0–2)
	Impact of CSU on job loss	0 (0–1)
	Impact of CSU on the need for early retirement	0 (0–0)
Life decisions related to education		
	Impact of CSU on academic performance	1 (0–2)
	Impact of CSU on achieving the desired educational level	0 (0–1)
Life decisions related to personal relationships		
	Impact of CSU on family relationships	2 (0.75–2.25)
	Impact of CSU on social relationships	2 (1–2.25)
	Impact of CSU on romantic relationships	1 (0–3)
	Impact of CSU on finding a desired partner	0 (0–0)
Life decisions related to reproductive desires and sexuality		
	Impact of CSU on the desire to have children	0 (0–0)
	Impact of CSU on having children	0 (0–0)
	Impact of CSU on having as many children as desired	0 (0–0)
	Impact of CSU on sexual problems	1 (0–2.25)
Life decisions related to housing		
	Impact of CSU on choosing a habitual residence	0 (0–1)
	Impact of CSU on choosing the location of the residence	0 (0–1)
	Impact of CSU on choosing to live abroad	0 (0–0.25)
Life decisions related to vacations and leisure		
	Impact of CSU on vacations and leisure within the country	2 (1–3)
	Impact of CSU on vacations and leisure abroad	2 (0–3)
Life decisions related to sports and lifestyle habits		
	Impact of CSU on lifestyle habits	2 (1–3)
	Impact of CSU on clothing choices	2 (1–3)
	Impact of CSU on sports participation	1 (1–3)
Life decisions related to substance use		
	Impact of CSU on smoking	1 (0–1)
	Impact of CSU on alcohol consumption	0 (0–1)
	Impact of CSU on the use of anxiolytics, antidepressants, or sleeping pills	1 (0–3)
	Impact of CSU on the use of other drugs	0 (0–0)
Life decisions related to symptoms		
	Impact of CSU on pain	2 (1–3)
	Impact of CSU on itching	3 (2.75–3)
	Impact of CSU on sleep deprivation	2.5 (0.75–3)
	Impact of CSU on fatigue	2 (0–3)
	Impact of CSU on shame	1 (0–2.25)
	Impact of CSU on confidence	1 (0–2)
	Impact of CSU on stress, anxiety or mood swings	2 (1–3)
	Impact of CSU on feeling sad	1 (0–3)

The variables in the table are expressed as median and interquartile range (IQR). Note: The list of MLCDs evaluated in this study was developed independently for the specific context of CSU and is not based on the MLCDP. While inspired by the conceptual framework introduced by Bhatti et al., our list aimed to address CSU-specific impacts and does not replicate or replace validated tools such as the MLCDP.

**Table 3:** Impact on Socio-Demographic Characteristics and MLCD by CSU disease activity.

Socio-Demographic Characteristics and MLCD by CSU Disease Activity.				
		Mild–Moderate CSU *n* = 9	Severe CSU *n* = 21	*p*-Value
Sex, % (*n*)				0.397
	Female	66.7 (6)	81 (17)	
	Male	33.3 (3)	19 (4)	
Age, mean (SD)		49.56 (18.72)	44.9 (12.82)	0.51
Age of disease onset, mean (SD)		38.33 (22.08)	41.71 (13.74)	0.679
Disease duration, mean (SD)		11.22 (16.79)	3.19 (3.28)	0.041 *
Employment status, % (*n*)				
	Student	22.2 (2)	0 (0)	0.033 *
	Active	44.4 (4)	90.5 (19)	
	Not active	11.1 (1)	4.8 (1)	
	Retired	22.2 (2)	4.8 (1)	
Education level, % (*n*)				1
	No education	0 (0)	0 (0)	
	Basic education	33.3 (3)	33.3 (7)	
	Higher education	66.7 (6)	66.7 (14)	
Smoking (yes) % (*n*)		22.2 (2)	42.9 (9)	0.282
Number of affected areas, mean (SD)		4.11 (1.54)	4.67 (1.62)	0.39
Most affected area, % (*n*)				
	Hands	55.6 (5)	57.1 (12)	0.936
	Genitalia	33.3 (3)	38.1 (8)	0.804
	Gluteal region	55.6 (5)	61.9 (13)	0.745
	Submammary	33.3 (3)	38.1 (8)	0.804
	Face	44.4 (4)	76.2 (16)	0.091
	Trunk and limbs	100 (9)	95.2 (20)	0.506
Comorbidities, % (*n*)				
	Anxiety	11.1 (1)	33.3 (7)	0.207
	Sleep disorders	33.3 (3)	42.9 (9)	0.626
	Atopic dermatitis	22.2 (2)	23.8 (5)	0.925
	Asthma	0 (0)	28.6 (6)	0.073
	Fatigue/Low performance	0 (0)	28.6 (6)	0.073
	Depression	11.1 (1)	19 (4)	0.593
	Rhinoconjunctivitis	0 (0)	23.8 (5)	0.109
Disease severity, mean (SD)				
NRS itch, mean (SD)		6.11 (2.52)	9.29 (0.9)	0.005 *
NRS sleep, mean (SD)		5.22 (2.91)	8.24 (2.1)	0.016 *
DLQI, mean (SD)		7.11 (4.7)	17.38 (6.22)	<0.001 *
Disease intrusiveness, % (*n*)				0.002 *
	Minimal	44.4 (4)	0 (0)	
	Moderate	44.4 (4)	23.8 (5)	
	High	0 (0)	38.1 (8)	
	Very high	11.1 (1)	38.1 (8)	
Current severity of CSU, % (*n*)				0.107
	Mild	55.6 (5)	19 (4)	
	Moderate	33.3 (3)	42.9 (9)	
	Severe	11.1 (1)	38.1 (8)	
Overall impact of CSU, % (*n*)				0.008 *
	Absent	11.1 (1)	0 (0)	
	Minimal	11.1 (1)	0 (0)	
	Moderate	44.4 (4)	4.8 (1)	
	High	22.2 (2)	52.4 (11)	
	Very high	11.1 (1)	42.9 (9)	
Vital decisions related to work, % (*n*)				
Career choice				0.03 *
	Not at all	50 (4)	47.4 (9)	
	Slightly	25 (2)	0 (0)	
	Moderately	25 (2)	10.5 (2)	
	Much	0 (0)	42.1 (8)	
Job performance				0.036 *
	Not at all	37.5 (3)	5.3 (1)	
	Slightly	25 (2)	10.5 (2)	
	Moderately	37.5 (3)	36.8 (7)	
	Much	0 (0)	47.4 (9)	
Promotion opportunities				0.124
	Not at all	62.5 (5)	26.3 (5)	
	Slightly	25 (2)	26.3 (5)	
	Moderately	12.5 (1)	5.3 (1)	
	Much	0 (0)	42.1 (8)	
Job absenteeism				0.237
	Not at all	25 (2)	10.5 (2)	
	Slightly	50 (4)	36.8 (7)	
	Moderately	25 (2)	15.8 (3)	
	Much	0 (0)	36.8 (7)	
Income and salary				0.373
	Not at all	75 (6)	42.1 (8)	
	Slightly	12.5 (1)	15.8 (3)	
	Moderately	12.5 (1)	21.1 (4)	
	Much	0 (0)	21.1 (4)	
Cause of job loss				0.409
	Not at all	87.5 (7)	57.9 (11)	
	Slightly	12.5 (1)	15.8 (3)	
	Moderately	0 (0)	5.3 (1)	
	Much	0 (0)	21.1 (4)	
Early retirement				0.577
	Not at all	100 (8)	78.9 (15)	
	Slightly	0 (0)	10.5 (2)	
	Moderately	0 (0)	5.3 (1)	
	Much	0 (0)	5.3 (1)	
Vital decisions related to education, % (*n*)				
Educational performance				0.22
	Not at all	75 (6)	35 (7)	
	Slightly	12.5 (1)	25 (5)	
	Moderately	12.5 (1)	15 (3)	
	Much	0 (0)	25 (5)	
Achieving desired educational level				0.038 *
	Not at all	100 (8)	40 (8)	
	Slightly	0 (0)	35 (7)	
	Moderately	0 (0)	15 (3)	
	Much	0 (0)	10 (2)	
Impact on personal relationships, % (*n*)				
Family relationships				0.022 *
	Not at all	44.4 (4)	14.3 (3)	
	Slightly	44.4 (4)	14.3 (3)	
	Moderately	0 (0)	42.9 (9)	
	Much	11.1 (1)	28.6 (6)	
Socializing with friends				0.486
	Not at all	33.3 (3)	14.3 (3)	
	Slightly	33.3 (3)	23.8 (5)	
	Moderately	22.2 (2)	33.3 (7)	
	Much	11.1 (1)	28.6 (6)	
Romantic relationship				0.147
	Not at all	77.8 (7)	33.3 (7)	
	Slightly	11.1 (1)	19 (4)	
	Moderately	0 (0)	14.3 (3)	
	Much	11.1 (1)	33.3 (7)	
Not having the desired partner				0.372
	Not at all	100 (9)	81 (17)	
	Slightly	0 (0)	9.5 (2)	
	Moderately	0 (0)	0 (0)	
	Much	0 (0)	9.5 (2)	
Impact on reproductive desire and sexuality, % (*n*)				
Desire to have children				0.094
	Not at all	77.8 (7)	81 (17)	
	Slightly	22.2 (2)	0 (0)	
	Moderately	0 (0)	9.5 (2)	
	Much	0 (0)	9.5 (2)	
Not having children				0.312
	Not at all	100 (9)	89.5 (17)	
	Slightly	0 (0)	0 (0)	
	Moderately	0 (0)	0 (0)	
	Much	0 (0)	10.5 (2)	
Not having all desired children				0.536
	Not at all	88.9 (8)	85 (17)	
	Slightly	11.1 (1)	5 (1)	
	Moderately	0 (0)	0 (0)	
	Much	0 (0)	10 (2)	
Having sexual problems				0.117
	Not at all	66.7 (6)	33.3 (7)	
	Slightly	22.2 (2)	9.5 (2)	
	Moderately	11.1 (1)	23.8 (5)	
	Much	0 (0)	33.3 (7)	
Impact on housing, % (*n*)				
Choice of usual housing				0.356
	Not at all	88.9 (8)	61.9 (13)	
	Slightly	0 (0)	19 (4)	
	Moderately	11.1 (1)	9.5 (2)	
	Much	0 (0)	9.5 (2)	
Choice of place to live				0.463
	Not at all	100 (9)	76.2 (16)	
	Slightly	0 (0)	14.3 (3)	
	Moderately	0 (0)	4.8 (1)	
	Much	0 (0)	4.8 (1)	
Living abroad				0.271
	Not at all	100 (9)	66.7 (14)	
	Slightly	0 (0)	14.3 (3)	
	Moderately	0 (0)	14.3 (3)	
	Much	0 (0)	4.8 (1)	
Impact on holidays and leisure, % (*n*)				
National holidays and leisure trips				0.106
	Not at all	33.3 (3)	9.5 (2)	
	Slightly	33.3 (3)	19 (4)	
	Moderately	33.3 (3)	33.3 (7)	
	Much	0 (0)	38.1 (8)	
International holidays and leisure trips				0.155
	Not at all	44.4 (4)	19 (4)	
	Slightly	22.2 (2)	14.3 (3)	
	Moderately	33.3 (3)	28.6 (6)	
	Much	0 (0)	38.1 (8)	
Impact on sports and lifestyle, % (*n*)				
Changing habits of lifestyle				0.002 *
	Not at all	33.3 (3)	4.8 (1)	
	Slightly	44.4 (4)	4.8 (1)	
	Moderately	22.2 (2)	38.1 (8)	
	Much	0 (0)	52.4 (11)	
Fashion choices				0.172
	Not at all	33.3 (3)	14.3 (3)	
	Slightly	33.3 (3)	14.3 (3)	
	Moderately	22.2 (2)	19 (4)	
	Much	11.1 (1)	52.4 (11)	
Sports practice				0.038 *
	Not at all	44.4 (4)	9.5 (2)	
	Slightly	44.4 (4)	28.6 (6)	
	Moderately	11.1 (1)	19 (4)	
	Much	0 (0)	42.9 (9)	
Impact on substance use, % (*n*)				
Smoking				0.118
	Not at all	0 (0)	55.6 (5)	
	Slightly	100 (2)	22.2 (2)	
	Moderately	0 (0)	0 (0)	
	Much	0 (0)	22.2 (2)	
Alcohol				0.348
	Not at all	71.4 (5)	37.5 (3)	
	Slightly	28.6 (2)	50 (4)	
	Moderately	0 (0)	12.5 (1)	
	Much	0 (0)	0 (0)	
Anxiolytics, antidepressants or sleeping pills				0.1
	Not at all	100 (3)	20 (2)	
	Slightly	0 (0)	30 (3)	
	Moderately	0 (0)	10 (1)	
	Much	0 (0)	40 (4)	
Other drugs				
	Not at all	100 (3)	100 (1)	
	Slightly	0 (0)	0 (0)	
	Moderately	0 (0)	0 (0)	
	Much	0 (0)	0 (0)	
Impact of symptoms, % (*n*)				
Pain				0.003 *
	Not at all	55.6 (5)	4.8 (1)	
	Slightly	33.3 (3)	19 (4)	
	Moderately	11.1 (1)	28.6 (6)	
	Much	0 (0)	47.6 (10)	
Itching				0.001 *
	Not at all	11.1 (1)	0 (0)	
	Slightly	0 (0)	0 (0)	
	Moderately	55.6 (5)	4.8 (1)	
	Much	33.3 (3)	95.2 (20)	
Sleep deprivation				0.002 *
	Not at all	66.7 (6)	4.8 (1)	
	Slightly	11.1 (1)	4.8 (1)	
	Moderately	11.1 (1)	23.8 (5)	
	Much	11.1 (1)	66.7 (14)	
Fatigue				0.002 *
	Not at all	77.8 (7)	9.5 (2)	
	Slightly	0 (0)	9.5 (2)	
	Moderately	22.2 (2)	42.9 (9)	
	Much	0 (0)	38.1 (8)	
Shame				0.01 *
	Not at all	55.6 (5)	19 (4)	
	Slightly	44.4 (4)	14.3 (3)	
	Moderately	0 (0)	33.3 (7)	
	Much	0 (0)	33.3 (7)	
Lack of confidence				0.086
	Not at all	55.6 (5)	33.3 (7)	
	Slightly	44.4 (4)	19 (4)	
	Moderately	0 (0)	23.8 (5)	
	Much	0 (0)	23.8 (5)	
Stress, anxiety or mood swings				0.01 *
	Not at all	44.4 (4)	4.8 (1)	
	Slightly	33.3 (3)	14.3 (3)	
	Moderately	0 (0)	38.1 (8)	
	Much	22.2 (2)	42.9 (9)	
Feeling sad				0.008 *
	Not at all	77.8 (7)	14.3 (3)	
	Slightly	11.1 (1)	23.8 (5)	
	Moderately	0 (0)	23.8 (5)	
	Much	11.1 (1)	38.1 (8)	

Quantitative variables are expressed as mean and standard deviation (SD) and categorical variables as relative (%) and absolute frequencies (*n*). * Statistically significant (*p* < 0.05).

## Data Availability

The data presented in this study are available on request from the corresponding author due to privacy and ethical restrictions. The data are not publicly available to protect the confidentiality of the participants in accordance with local regulations and ethics committee mandates.

## Abbreviations

The following abbreviations are used in this manuscript:

AD	Atopic dermatitis
CSU	Chronic Spontaneous urticaria
DLQI	Dermatology Life Quality Index
HRQoL	Health-related quality of life
IQR	Interquartile range
MLCD	Major life-changing decisions
MLCDP	Major Life-Changing Decision Profile
NRS	Numerical Rating Scale
PROMs	Patient-reported outcome measures
SD	Standard deviation
SPSS	Statistical Package for the Social Sciences
UAS7	Urticaria Activity Score over 7 days

## Appendix A. Sample Patient Data Collection Questionnaire for UC

A rellenar por el dermatólogo					
Edad:					
Sexo:	__Hombre.	__Mujer			
Edad de debut de la enfermedad:					
Situación laboral.	__Estudiante,	__Activo,	___No Activo,	__Jubilado	
Nivel de estudios:	__Sin estudios;	__Básicos;	__Superiores		
Número de zonas afectas:					
Hábito tabáquico:	__Si	__No			
Qué zona es la más afectada					
__Mano,	___Genitales,	__Glúteos,	__Submamaria,	__Cara,	__Tronco y extremidades
COMORBILIDADES: (MARCAR LAS QUE SÍ)					
__Ansiedad					
__Depresión					
__Dermatitis atópica					
__Asma					
__Rinoconjuntivitis					
__Bajo rendimiento					
__Problemas del sueño					
UAS7:					
DLQI:					
NRS picor (0–10):					
NRS sueño (0–10):					
Intrusividad de la enfermedad en la vida del paciente (subjetivo en base a la anamnesis)					
__Ausente,	__Mínima,	__Moderada,	__Elevada,	__Muy elevada	
A rellenar por el paciente					
¿Cómo consideras que es la gravedad de tu URTICARIA CRÓNICA en el momento actual?					
__Leve					
__Moderada					
__Grave					
¿De forma global cómo consideras que ha sido el impacto de la URTICARIA CRÓNICA a lo largo de tu vida?					
__Ausente					
__Mínimo					
__Moderado					
__Elevado					
__Muy elevado					
IMPACTO A NIVEL PROFESIONAL:					
LA URTICARIA CRÓNICA A LO LARGO DE MI VIDA HA INFLUENCIADO…					
Nunca he trabajado salta siguiente sección					
La elección de carrera profesional/ocupación					
__En absoluto;	__Levemente;	__Moderadamente:	__Mucho		
Mi desempeño laboral (lo bien que hago mi trabajo)					
__En absoluto;	__Levemente;	__Moderadamente:	__Mucho		
Mis oportunidades de ascenso o promoción laboral o de progresión de mi negocio					
__En absoluto;	__Levemente;	__Moderadamente:	__Mucho		
Los días que he tenido que faltar a mi trabajo					
__En absoluto;	__Levemente;	__Moderadamente:	__Mucho		
Mis ingresos/salario					
__En absoluto;	__Levemente;	__Moderadamente:	__Mucho		
Ha sido causa directa de perder/dejar mi trabajo					
__En absoluto;	__Levemente;	__Moderadamente:	__Mucho		
Ha motivado mi jubilación prematura					
__En absoluto;	__Levemente;	__Moderadamente:	__Mucho		
EDUCACIÓN:					
LA URTICARIA CRÓNICA A LO LARGO DE MI VIDA HA INFLUENCIADO…					
Mi rendimiento educativo (Lo buen/a estudiante que he sido)					
__En absoluto;	__Levemente;	__Moderadamente:	__Mucho		
Alcanzar el nivel educativo deseado por mí (secundaria, bachiller, FP, universitario etc.…)					
__En absoluto;	__Levemente;	__Moderadamente:	__Mucho		
RELACIONES PERSONALES					
LA URTICARIA CRÓNICA A LO LARGO DE MI VIDA HA INFLUENCIADO					
Mis relaciones familiares					
__En absoluto;	__Levemente;	__Moderadamente:	__Mucho		
Mi nivel de socialización con otras personas, los/as amigos/as que tengo					
__En absoluto;	__Levemente;	__Moderadamente:	__Mucho		
Mi vida sentimental					
__En absoluto;	__Levemente;	__Moderadamente:	__Mucho		
No tener la pareja que me hubiese gustado					
__En absoluto;	__Levemente;	__Moderadamente:	__Mucho		
DESEOS REPRODUCTIVOS Y SEXUALIDAD					
LA URTICARIA CRÓNICA A LO LARGO DE MI VIDA HA INFLUENCIADO					
Mi deseo de tener hijo/as					
__En absoluto;	__Levemente;	__Moderadamente:	__Mucho		
Si todavía no se ha planteado tener hijo/as pase a la siguiente sección					
No haber tenido hijo/as					
__En absoluto;	__Levemente;	__Moderadamente:	__Mucho		
No haber tenido todos los hijos/as que hubiera deseado					
__En absoluto;	__Levemente;	__Moderadamente:	__Mucho		
Tener problemas sexuales					
__En absoluto;	__Levemente;	__Moderadamente:	__Mucho		
VIVIENDA					
LA URTICARIA CRÓNICA A LO LARGO DE MI VIDA HA INFLUENCIADO					
La elección de mi vivienda habitual (su ubicación, sus características)					
__En absoluto;	__Levemente;	__Moderadamente:	__Mucho		
La elección de la localidad donde vivo					
__En absoluto;	__Levemente;	__Moderadamente:	__Mucho		
Vivir en el extranjero					
__En absoluto;	__Levemente;	__Moderadamente:	__Mucho		
VACACIONES Y OCIO					
LA URTICARIA CRÓNICA A LO LARGO DE MI VIDA HA INFLUENCIADO					
Mis vacaciones y viajes de ocio nacionales (la frecuencia, el destino o la duración)					
__En absoluto;	__Levemente;	__Moderadamente:	__Mucho		
Mis vacaciones y viajes de ocio al extranjero (la frecuencia, el destino o la duración)					
__En absoluto;	__Levemente;	__Moderadamente:	__Mucho		
DEPORTE Y HÁBITOS DE VIDA					
LA URTICARIA CRÓNICA A LO LARGO DE MI VIDA HA INFLUENCIADO					
Cambiar hábitos o mi estilo de vida por mi problema de salud					
__En absoluto;	__Levemente;	__Moderadamente:	__Mucho		
Mi forma de vestir					
__En absoluto;	__Levemente;	__Moderadamente:	__Mucho		
La práctica de deporte (el tipo o la frecuencia)					
__En absoluto;	__Levemente;	__Moderadamente:	__Mucho		
HÁBITOS TÓXICOS					
LA URTICARIA CRÓNICA A LO LARGO DE MI VIDA HA INFLUENCIADO					
El tabaco que fumo					
__En absoluto;	__Levemente;	__Moderadamente:	__Mucho		
Las bebidas alcohólicas que consumo					
__En absoluto;	__Levemente;	__Moderadamente:	__Mucho		
Los ansiolíticos, antidepresivos o medicamentos que tomo para dormir					
__En absoluto;	__Levemente;	__Moderadamente:	__Mucho		
El consumo de drogas					
__En absoluto;	__Levemente;	__Moderadamente:	__Mucho		
¿Hay algo que consideres que haya disminuido el efecto negativo que la URTICARIA CRÓNICA ha tenido en tu vida? Por ejemplo, algún tratamiento, cirugía, haber perdido peso, haberte hecho la depilación láser					
Descríbelo:					
INDICA EN QUÉ MEDIDA ESTOS SÍNTOMAS HAN INFLUENCIADO NEGATIVAMENTE TU VIDA					
El dolor					
__En absoluto;	__Levemente;	__Moderadamente:	__Mucho		
El picor					
__En absoluto;	__Levemente;	__Moderadamente:	__Mucho		
La falta de sueño					
__En absoluto;	__Levemente;	__Moderadamente:	__Mucho		
Limitación física					
__En absoluto;	__Levemente;	__Moderadamente:	__Mucho		
Vergüenza					
__En absoluto;	__Levemente;	__Moderadamente:	__Mucho		
Falta de confianza					
__En absoluto;	__Levemente;	__Moderadamente:	__Mucho		
Estrés miedo o ansiedad					
__En absoluto;	__Levemente;	__Moderadamente:	__Mucho		
Deprimirme y estar triste					
__En absoluto;	__Levemente;	__Moderadamente:	__Mucho		
